# Simultaneous Determination of Five Components in Rat Plasma by UPLC–MS/MS and Its Application to a Comparative Pharmacokinetic Study in Baihe Zhimu Tang and Zhimu Extract

**DOI:** 10.3390/molecules20046700

**Published:** 2015-04-15

**Authors:** Guolong Li, Zhishu Tang, Jie Yang, Jinao Duan, Dawei Qian, Jianming Guo, Zhenhua Zhu, Hongbo Liu

**Affiliations:** 1 Shaanxi Collaborative Innovation Center of Chinese Medicinal Resource Industrialization, Shaanxi University of Chinese Medicine, Xianyang 712046, China; 2 Jiangsu Collaborative Innovation Center of Chinese Medicinal Resources Industrialization, and National and Local Collaborative Engineering Center of Chinese Medicinal Resources Industrialization and Formulae Innovative Medicine, Nanjing University of Chinese Medicine, Nanjing 210023, China

**Keywords:** compatibility, Baihe Zhimu Tang, pharmacokinetics

## Abstract

Baihe Zhimu Tang (BZT) is a famous traditional Chinese medicine recipe to treat dry coughing due to yin deficiency and for moisturizing the lungs. Zhimu is an essential ingredient in BZT used to treat inflammation, fever and diabetes. The most important active components in Zhimu are flavonoids such as neomangiferin, mangiferin, and steroid saponins (e.g., timosaponin BII, anemarsaponin BIII, timosaponin AIII). The aim of this study was to compare the pharmacokinetics of mangiferin, neomangiferin, timosaponin BII, anemarsaponin BIII and timosaponin AIII in rat plasma after oral administration of BZT and Zhimu extract (ZME). A sensitive, reliable and robust LC-MS/MS method to simultaneously determine steroid saponins and flavonoids in rat plasma was successfully validated. Significant differences (*p* < 0.05) were found in the pharmacokinetic parameters of timosaponin BII, anemarsaponin BIII and timosaponin AIII between BZT and ZME. It was surmised that formula compatibility could significantly influence the pharmacokinetics of BZT and our study is the first to study the administration of BZT based on pharmacokinetic studies.

## 1. Introduction

Traditional Chinese medicines (TCMs) have been widely used for the prevention and treatment of various diseases for thousands of years in China. Decoctions (tang in Chinese), or combinations of one or more TCM herbs, are frequently used as basic composition units of Chinese herbal formulas for achieving mutual reinforcement and decrease adverse side effects. Consequently, the compatibility of Chinese medicinal herbs is a significant theory in the combination of TCM. Overall, the law of compound compatibility is studied through pharmacodynamics [1] and pharmacokinetics [2,3]. A pharmacokinetic method was used in this study, which could help understand the mechanism of action of drugs and the compatible priority of the whole prescription. 

Baihe Zhimu Tang (BZT), containing Baihe (*Bulbus Lilii*, the dried bulbs of *Lilium lancifolum* Thunb.) and Zhimu (*Rhizoma Anemarrhenae*, the dried rhizome of *Anemarrhena asphodeloides* Bge.), is a famous traditional Chinese medicine recipe, firstly described in ‘Jin gui Yao lue’, a well-known formula book edited by the Chinese physician Zhang Zhongjing (150–219 A.D. in the Chinese Eastern Han Dynasty) [4]. BZT has been frequently used for stopping dry coughing due to yin deficiency and moisturizing the lungs, clearing away the heat evil in the pericardium and calming the nerves, treating diabetes due to internal heat and constipation, *etc.* [5,6,7].

**Figure 1:**
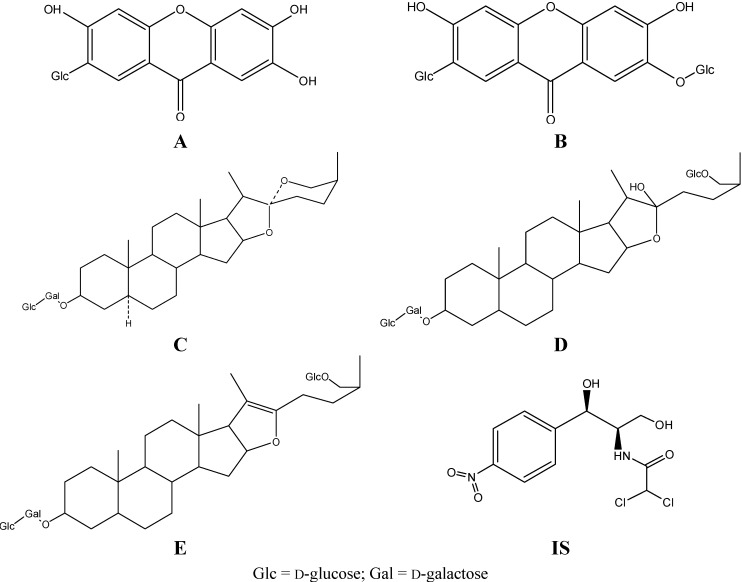
Chemical structures of mangiferin (**A**), neomangiferin (**B**), timosaponin BII (**C**) anemarsaponin BIII (**D**) and timosaponin AIII (**E**) and the internal standard chloramphenicol (**IS**).

Zhimu is used as an essential ingredient in BZT to treat inflammation, fever and diabetes [7,8]. Modern pharmacology studies showed that *Anemarrhena asphodeloides* possesses antitumor, antiviral, immunomodulatory and antioxidant activities [9,10,11]. Flavonoids such as mangiferin, neomangiferin and steroid saponins (e.g., timosaponin BII, anemarsaponin BIII, timosaponin AIII, Figure 1) are the most important active components in *Anemarrhena asphodeloides* [5,12]. Timosaponin BII and mangiferin are used as phytochemical markers for the quality control of *A. asphodeloides* in the Chinese Pharmacopeia [7]. There were some reports that compare the pharmacokinetics of timosaponin BII and timosaponin AIII after oral administration of Zhimu–Baihe herb-pair and Zhimu extract in rats [13], to compare the pharmacokinetic profiles of timosaponin BII, timosaponin AIII, mangiferin and neomangiferin after oral administration of Er-MU preparation and Zhimu extract [14] and to compare the pharmacokinetics of timosaponin BII and timosaponin AIII after oral administration of Zhimu–Huangbai herb-pair [15], but there are no reported studies of the pharmacokinetics of neomangiferin, mangiferin and anemarsaponin BIII in BZT in comparison with Zhimu extract (ZME).

In the present study, a sensitive, reliable and robust LC-MS/MS method was developed to determine the concentrations of mangiferin, neomangiferin, timosaponin BII, anemarsaponin BIII, and timosaponin AIII in rat plasma after the intragastic administration of BZT and ZME, repectively. The pharmacokinetic parameters and pharmacokinetics of the five ingredients in rat plasma after oral the administration BZT and ZME were different. The results of our study should help us to explore whether there are synergistic effects between the five ingredients in BZT. At the same time, these methods may contribute solutions to study the reasonableness of the Chinese herbal compound. 

## 2. Results and Discussion

### 2.1. Optimization of the Chromatographic Conditions

In order to optimize the MS conditions, both positive and negative scan modes were evaluated. Mangiferin, neomangiferin, timosaponin BII, anemarsaponin BIII and timosaponin AIII responded better in negative ion mode compared with positive ion mode. With respect to the mobile phase, it was recommended that the analysis of furostanol saponins such as timosaponin BII, by LC–MS be performed using aqueous acetonitrile mobile phase but not methanol due to the interconversion of the C-22 hydroxy and C-22 methoxy forms [16], hence acetonitrile was chosen as the organic phase. Moreover, the use of 0.1% formic acid in the water phase could improve the ionization efficiency of the analytes and decrease the response intensity of the endogenous matrix. Therefore acetonitrile and 0.1% formic acid as the mobile phase with gradient elution was applied, giving favorable retention times and low background noise.

### 2.2. Sample Preparation

In order to obtain higher recovery of the analytes and IS and less endogenous interference two for sample preparation methods, including liquid-liquid extraction (LLE) and protein precipitation, were tested and compared. However, after application of different extraction solvents (ethyl acetate, dichloromethane, *n*-butanol), the recovery of less than 50% for LLE was not satisfactory, so a feasible protein precipitation procedure with acetonitrile was used. Although an obvious matrix effect was observed with this protein precipitation method compared to other sample preparation methods, the protein precipitation is a simple and fast method, so it was used as the sample preparation procedure.

### 2.3. Method Validation

#### 2.3.1. Selectivity

**Figure 2:**
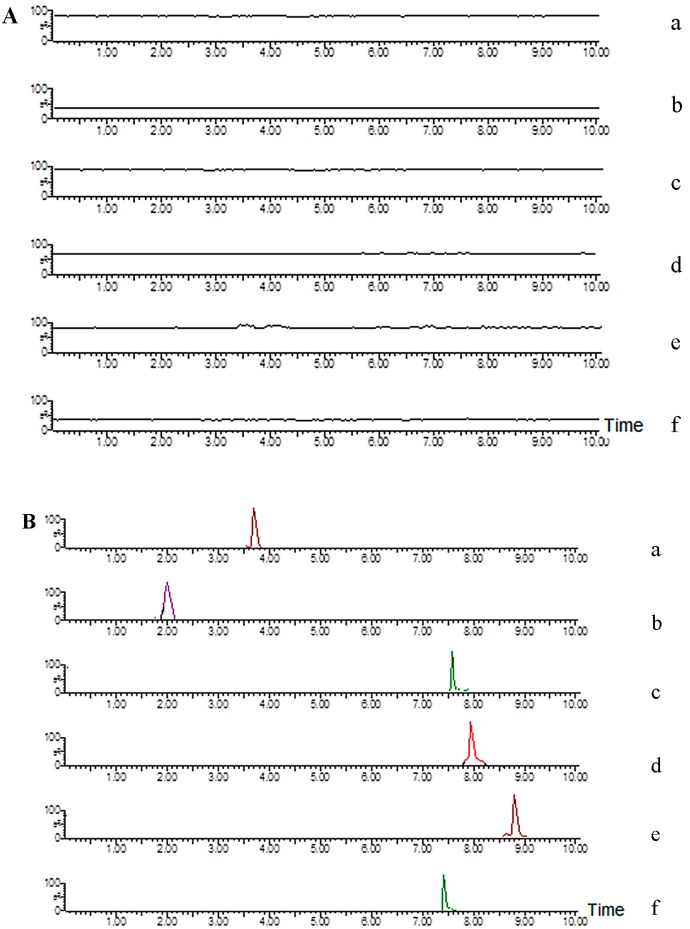

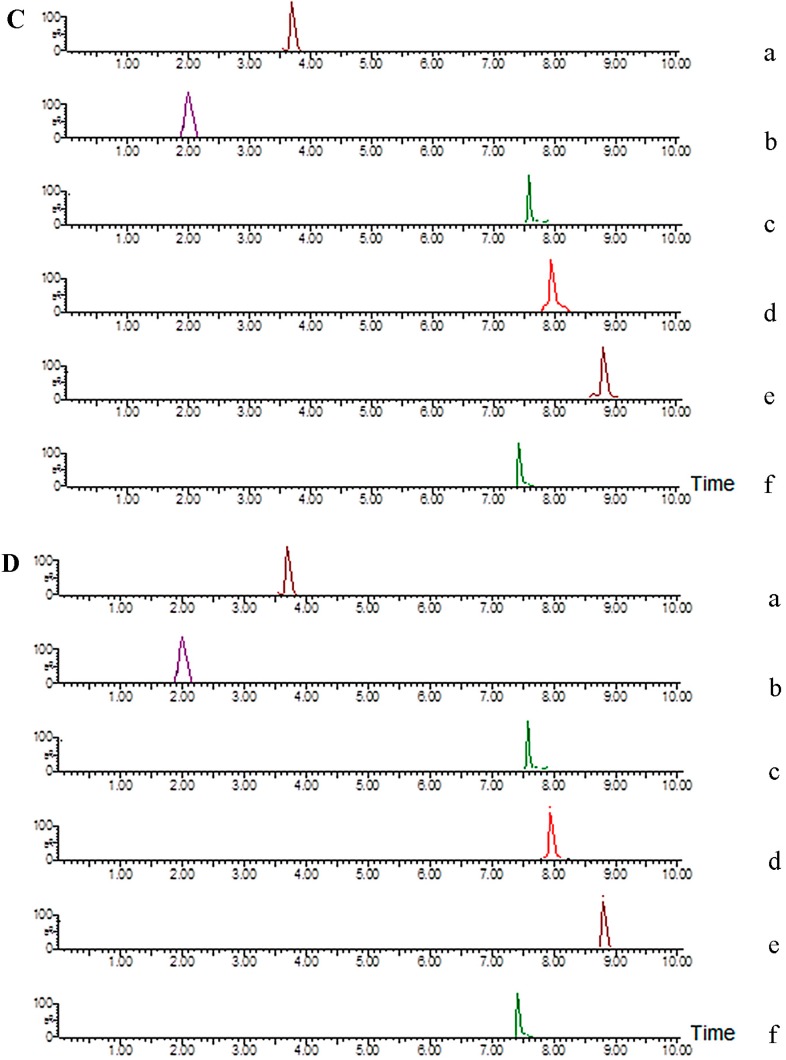
MRM chromatograms of each compound (**a**. mangiferin; **b**. neomangiferin; **c**. timosaponin BII; **d**. anemarsaponin BIII; **e**. timosaponin AIII; **f**. chloramphenicol). (**A**) blank plasma; (**B**) blank plasma spiked with each compound at LLOQ; (**C**) plasma sample at 45 min after oral BZT; (**D**) plasma sample at 45 min after oral ZME.

Figure 2 shows the representative MRM chromatograms of the blank rat plasma (A), blank rat plasma spiked with five analytes and IS (B), and an *in vivo* plasma sample obtained 45 min after oral administration of BZT (C). 

The retention times were about 1.80, 3.56, 7.45, 7.70, 8.65 and 7.40 min for neomangiferin, mangiferin, timosaponin BII, anemarsaponin BIII, timosaponin AIII and chloramphenicol, respectively. No endogenous peaks were observed in the representative chromatograph of blank plasma sample at the retention times of the analytes and IS.

#### 2.3.2. Linearity and LLOQ

Representative calibration curves were as follows: *Y* = 0.0074*X* − 0.028 (r = 0.9989, mangiferin), *Y* = 0.001*X* + 0.016 (r = 0.9976, neomangiferin), *Y* = 0.002*X* + 0.004 (r = 0.9984, timosaponin BII), *Y* = 0.0009*X* + 0.023 (r = 0.9978, anemarsaponin BIII), *Y* = 0.004*X* − 0.006 (r = 0.9994, timosaponin AIII). The lowest concentrations with RSD < 20% were taken as LLOQs and were found to be 8.06 ng/mL for mangiferin, 10.3 ng/mL for neomangiferin, 9.05 ng/mL for timosaponin BII, 6.3 ng/mL for anemarsaponin BIII, 6.25 ng/mL for timosaponin AIII, respectively, which were sufficient for pharmacokinetic studies.

#### 2.3.3. Accuracy and Precision

Accuracy and intra- and inter-day of LLOQ and three QC samples are presented in Table 1. The accuracy data in the present study ranged from 87.5% to 109.8% (RE), and the intra- and inter-day precision were 3.6% to 10.4% and 4.9% to 11.8% (RSD), respectively. The results indicated that the precision and accuracy values were acceptable.

**Table 1:** The accuracy and intra- and inter- day precision of five analytes in rat plasma (n = 5).

Analytes	Concentration ng/mL	Intra-Day		Inter-Day	
		Accuracy (%)	Precision (RSD, %)	Accuracy (%)	Precision (RSD, %)
Mangiferin	10.32	92.6	9.1	102.0	10.9
	103.2	91.3	10.4	95.9	9.0
	1032	93.1	8.3	95.4	8.1
	8.06	99.2	7.4	104.1	8.6
Neomangiferin	13.2	91.5	7.2	99.6	11.8
	132.8	99.4	6.1	102.4	10.9
	1328	101.5	6.8	99.8	10.6
	10.3	99.5	5.9	103.8	8.6
Timosaponin BII	11.6	87.8	7.1	94.9	11.7
	116	87.5	6.7	91.1	9.8
	1160	103.9	8.4	102.6	11.1
	9.05	109.8	5.2	108.0	7.4
Anemarsaponin BIII	9.2	100.4	5.7	101.7	8.7
	92	105.8	3.6	103.6	8.8
	920	101.5	5.6	99.1	8.4
	6.3	103.4	5.0	102.2	6.8
Timosaponin AIII	8.8	92.0	6.9	99.7	10.8
	88.8	93.4	4.2	104.3	9.5
	888	97.2	5.4	103.8	10.1
	6.25	103.5	5.9	103.8	4.9

#### 2.3.4. Extraction Recovery and Matrix Effect

The mean recoveries and matrix effect was evaluated by analyzing QC samples at three concentrations with five replicates. As detailed in Table 2, the mean recovery of the analytes was within 72.9%–101.8% (RSD < 15%). The extraction recovery of IS was 78.2% ± 8.3%. The corresponding matrix effect ranged from 83.2% to 102.7% (RSD < 15%). The matrix effect of IS was 85.4% ± 4.2%. Thus, it was manifested that acetonitrile was a feasible and appropriate medium for the extraction of the analytes and IS, and moreover, there was no measurable matrix effect on the ionization of analytes and IS.

**Table 2:** Recovery and matrix effects for five analytes in rat plasma (n = 5).

Analytes	Concentration (ng/mL)	Recovery		Matrix Effects	
		Accuracy (%)	Precision (%)	Accuracy (%)	Precision (RSD, %)
Mangiferin	10.32	76.9	7.6	96.3	4.1
	103.2	72.9	5.8	109.8	10.9
	1032	78.8	9.6	96.2	7.0
Neomangiferin	13.2	78.8	7.4	91.1	8.4
	132.8	74.5	1.2	98.6	9.9
	1328	75.3	7.3	100.6	9.4
Timosaponin BII	11.6	96.4	11.9	99.0	10.4
	116	88.8	5.5	88.8	8.3
	1160	89.4	7.3	87.7	8.1
Anemarsaponin BIII	9.2	89.3	11.5	88.9	9.1
	92	101.8	12.3	93.9	5.9
	920	91.4	10.1	96.2	7.7
Timosaponin AIII	8.8	92.0	7.9	94.5	7.9
	88.8	82.1	9.9	87.8	6.5
	888	98.6	7.9	95.7	9.6

#### 2.3.5. Sample Stability

The stability of QC samples at three concentrations under different conditions was evaluated based on peak areas in comparison with freshly prepared QC samples. The results are presented in Table 3. The results indicated that these analytes were all stable in rat plasma after one-month storage at −80 °C, 24 h in the auto-sampler (4 °C) and three freeze-thaw cycles with accuracy in the range from 81.3% to 104.9%. 

**Table 3:** Stability for five analytes in rat plasma (n = 5).

Analytes	Concentration (ng/mL)	Auto-sampler for 24 h		At −80 °C for 1 month		Freeze-Thaw Cycles	
		Mean (%)	RSD (%)	Mean (%)	RSD (%)	Mean (%)	RSD (%)
Mangiferin	10.32	95.2	8.7	90.3	4.7	97.1	11.7
	103.2	99.7	10.8	104.9	10.5	101.6	8.6
	1032	98.1	9.9	84.3	12.7	97.7	10.1
Neomangiferin	13.2	84.5	9.2	84.7	4.8	91.7	12.5
	132.8	89.0	4.3	84.0	12.3	97.5	9.2
	1328	95.5	8.6	81.3	13.1	92.5	9.5
Timosaponin BII	11.6	98.6	9.2	100.2	10.4	91.4	9.2
	116	83.2	4.1	88.8	5.5	84.6	9.5
	1160	90.5	9.9	95.6	5.6	93.8	12.7
Anemarsaponin BIII	9.2	92.6	12.0	96.3	10.3	102.2	11.3
	92	101.9	5.4	101.8	7.0	97.1	8.2
	920	98.4	5.3	103.7	5.4	100.3	3.8
Timosaponin AIII	8.8	102.7	6.2	96.6	6.3	97.3	11.7
	88.8	88.7	4.6	93.6	12.1	87.6	8.0
	888	101.2	11.0	98.0	7.8	100.1	12.1

### 2.4. Pharmacokinetic Study

**Figure 3:**
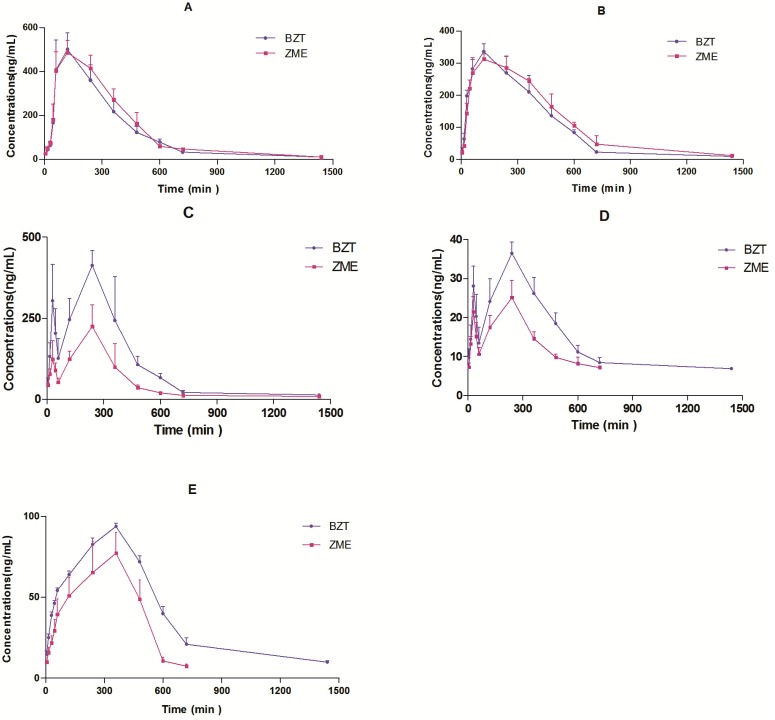
Mean concentration-time profiles of mangiferin (**A**), neomangiferin (**B**), timosaponin BII (**C**), anemarsaponin BIII (**D**) and timosaponin AIII (**E**) in rat plasma after oral administration of BZT and ZME. Each point represents the mean ± SD (n = 6).

The validated method was successfully applied to a comparative pharmacokinetic study of mangiferin, neomangiferin, timosaponin BII, anemarsaponin BIII, and timosaponin AIII in rat plasma after the oral administration of BZT and ZME. The mean plasma concentration-time profiles are shown in Figure 3, and the pharmacokinetic parameters are presented in Table 4.

When BZT was administered orally to rats, the AUC of timosaponin BII, anemarsaponin BIII and timosaponin AIII of BZT were increased significantly. It was demonstrated that the Zhimu compatibility of Baihe could increase the extent of bioavailability of steroid saponins without increasing C_max_ and prolonging *T*_1/2_. The reasons need further study.

**Table 4:** Pharmacokinetics parameters of analytes in male SD rat following oral administration of BZT and ZME (mean ± SD, n = 6).

Parameters	Mangiferin		Neomangiferin	
	BZT	ZME	BZT	ZME
C_max_ (ng/mL)	522.83 ± 53.3	486.7 ± 20.1	336.7 ± 24.5	318.0 ± 10.5
T_max_ (min)	96.0 ± 32.8	132.0 ± 65.7	120	144 ± 53.7
T_1/2_ (min)	285.92 ± 13.6	269.5 ± 165.4	258.2 ± 24.4	261.9 ± 34.4
AUC_0-t_ (ng/L min）	175,960.7 ± 21,321.91	190,276.9 ± 12,608.0	145,297 ± 8204.4	164,643 ± 13,198.7
MRT_0-t_ (min)	331.8 ± 14.5	312.1 ± 4.6	324.4 ± 11.5	365.4 ± 34.7
Parameters	**Timosaponin BII**		**Anemarsaponin BIII**	
	BZT	ZME	BZT	ZME
C_max_ (ng/mL)	434.6 ± 33.0 *	239.5 ± 47.6	36.4 ± 2.8 *	25.0 ± 4.5
T_max_ (min)	264 ± 53.6	240 ± 84.8	240	264 ± 53.6
T_1/2_ (min)	304.6 ± 136.3 *	161.7 ± 44.3	299.8 ± 128.4 *	197.7 ± 60.4
AUC_0-t_ (ng/L min）	150,045.5 ± 20,160.6 *	70,668.9 ± 11,834.0	17,531.5 ± 3348.7 *	9766.9 ± 1345.5
MRT_0-t_ (min)	333.6 ± 13.9	331.9 ± 39.3	289.0 ± 22.2	389.7 ± 106.5
Parameters	**Timosaponin AIII**			
	BZT	ZME		
C_max_ (ng/mL)	94.4 ± 3.8	84.3 ± 8.6		
T_max_ (min)	336.0 ± 53.7	312 ± 65.7		
T_1/2_ (min)	211.8 ± 98.8 *	104.2 ± 10.3		
AUC_0-t_ (ng/L min）	56,464.5 ± 7189.6 *	31,753.7 ± 3134.9		
MRT_0-t_ (min)	454.9 ± 23.2 *	308.1 ± 16.8		

Data are expressed as mean ± S.D. (n = 6); * Difference from corresponding Group ZME, *p <* 0.05.

There was no statistically significant difference in the pharmacokinetic parameters of mangiferin and neomangiferin between BZT and ZME. It was demonstrated that Baihe did not influence the bioavailability of mangiferin and neomangiferin.

The plasma concentration-time curves of timosaponin BII and timosaponin BIII in ZME after oral administration showed a double peak. The plasma concentration-time curves of timosaponin BII and timosaponin BIII in BZT after oral administration still showed a double peak. This phenomenon was also reported when Zhimu single extract was used or the compatibility of other medicines was studied. It indicated that Baihe increased the extent of bioavailability of timosaponin BII, timosaponin BIII only by increasing *C*_max_ and prolonging *T*_1/2_ [17,18]. No changes in their absorption process in the gastrointestinal tract were observed.

## 3. Experimental

### 3.1. Chemicals and Reagents

The reference standards of timosaponin BII (>98% purity) and neomangiferin (>98% purity) were purchased from Chengdu Herb Purify Co., Ltd. (Chengdu, China). Anemarsaponin BIII (>98% purity) and timosaponin AIII (>98% purity) were obtained from Chengdu Must Bio-technology Co., Ltd. (Chengdu, China). Mangiferin was purchased from Nanjing Saiyan Biology Co., Ltd. (Chengdu, China). Chloromycetin was purchased from the Chinese National Institute of Pharmaceutical and Biological Products (Beijing, China). The acetonitrile and formic acid of HPLC grade were purchased from Merck (Darmstadt, Germany). Deionized water was purified by an EPED water purification system (EPED, Nanjing, China). All other reagents used were of analytical grade. Zhimu [*A. asphodeloides* Bge. (Fam. Liliaceae)] and Baihe [*L. brownii* var. *viridulum* (Fam. Liliaceae)] were collected from Anhui Province. The Zhimu and Baihe samples were deposited in our laboratory.

### 3.2. UPLC–MS/MS Instrumentation and Conditions

Chromatographic analysis was performed on an Acquity UPLC system (Waters Corp., Milford, MA, USA), consisting of a binary pump solvent management system, an online degasser, and an autosampler. An Acquity UPLC BEH C18 column (100 mm × 2.1 mm, 1.7 µm) was employed and the column temperature was maintained at 35 °C. The mobile phase was composed of A (0.1% formic acid) and B (acetonitrile) using a gradient elution of 9%–9% B at 0–4.5 min, 9%–20% B at 4.5–5.5 min, 20%–25% B at 5.5–6.5 min, 25%–99% B at 6.5–8.5 min, 99%–9% B at 8.5–10 min a flow rate set at 0.4 mL/min. The autosampler was conditioned at 4 °C and the injection volume was 2 μL. 

Mass spectrometry analysis was performed using a Xevo TM triple quadrupole mass spectrometer (Waters Corp.) equipped with an electrospray ionization source (ESI). The ESI source was set in negative ionization mode. The parameters in the source were set as follows: capillary voltage 3.0 kV; source temperature 150 °C; Desolvation temperature 550 °C; cone gas flow 50 L/h; desolvation gas flow 1000 L/h. Analytes were performed by using multiple-reaction monitoring (MRM) mode employing the following precursor-to-product ion pair: 421.2→301.0 for mangiferin, 583.1→331.0 for neomangifer, 919.5→757.4 for timosaponin BII, 901.5→739.4 for anemarsaponin BIII, 739.4→577.4 for timosaponin AIII and 321.1→151.7 for chloromycetin. All data collected in centroid mode were acquired using Masslynx4.1 software (Waters Corp.) and post-acquisition quantitative analysis was performed using the Targetlynx program (Waters Corp).

### 3.3. Preparation of Zhimu Baihe Tang and Zhimu Extract

*L. brownii* (Baihe) and *A. asphodeloides* (Zhimu) were chopped into slices before use. The mixture of Baihe (300 g) and Zhimu (100 g) was immersed in 4 L of water and extracted two times by refluxing with boiling water for 2 h. After filtration through four layers of gauze, the supernatant was condensed to 400 mL under reduced pressure to obtain Baihe Zhimu Tang (BZT). The same Zhimu (100 g) was immersed in 1 L water and extracted two times by refluxing with boiling water for 2 h. After filtration through four layers of gauze, the supernatant was condensed to 100 mL under reduced pressure to obtain Zhimu extract (ZME). The BZT and ZME were stored at −20 °C before usage. To calculate the administration dose, the contents of five components in the decoctions were quantitatively determined by the external standard method with the same chromatography conditions as described in Section 3.2. The contents of neomangifer, mangiferin, timosaponin BII, anemarsaponin BIII, and timosaponin AIII were 6.55, 2.51, 10.37, 0.78 and 1.92 mg/mL and 6.51, 2.49, 10.31, 0.79 and 1.87 mg/mL in BZT and ZME, respectively. The relative deviation was applied to compare the differences of values between two groups. The relative deviations of mangifer, neomangiferin, timosaponin BII, anemarsaponin BIII, and timosaponin AIII were 0.61%, 0.80%, 0.58%, 1.26% and 2.67%, respectively. All of them were below 3%, which could implied that there were no differences in the content of compounds between BZT and ZME samples.

### 3.4. Preparation of Calibration Standards, Quality Control and Internal Standard

The stock solutions of mangiferin, neomangiferin, timosaponin BII, anemarsaponin BIII, timosaponin AIII and IS were prepared by dissolving them separately in 50% acetonitrile. A series of working standard solutions were prepared by dilutions of the stock solution with 50% acetonitrile to obtain the following concentrations: 12,900, 6450, 3225, 1290, 645, 322.5, 161.2, 80.6 ng/mL for mangiferin; 16,600, 8300, 4150, 1660, 830.0, 415.0, 207.5 and 103.7 ng/mL for neomangiferin; 14,500, 7250, 3625, 1450, 725, 362.5, 181.2 and 90.6 ng/mL for timosaponin BII; 11,500, 5750, 2875, 1150, 575, 287.5, 143.7 and 71.8 ng/mL for anemarsaponin BIII; 11,100, 5550, 2775, 1110, 555, 277.5, 138.7 and 69.3 ng/mL for timosaponin AIII and IS (3.8 μg/mL for chloramphenicol), respectively. Quality control (QC) samples at 103, 1032, 10320 ng/mL for mangiferin, 133, 1328, 13280 ng/mL for neomangiferin, 116, 1160, 11,600 ng/mL for timosaponin BII, 92, 920, 9200 ng/mL for anemarsaponin BIII, 88, 888, 8880 ng/mL for timosaponin AIII. All solutions were stored at 4 °C before analysis.

### 3.5. Sample Preparation

Frozen plasma samples were unfrozen at room temperature and treated as follows: to each 200 µL plasma sample, 10 µL of IS, 10 µL acetonitrile–water (1:1, *v*/*v*) or 10 µL of the working solution for the calibration curve and 600 µL acetonitrile were added. After vortex for 2 min and centrifugation at 10,400× *g* at 4 °C for 10 min The supernatant was transferred into a new tube and evaporated to dryness in a rotary evaporator at 39 °C and the residue was reconstituted in 100 µL of 0.1% formic acid-acetonitrile (50:50, *v*/*v*), vortex for 2 min and centrifugation at 10,400× *g* at 4 °C for 10 min. The supernatant was transferred to an auto sampler vial and an aliquot of 2 μL was injected onto the UPLC–MS/MS system for analysis.

### 3.6. Method Validation

The method was validated in terms of selectively, linearity, LLOQ, precision, accuracy, recovery, matrix effect and stability of the analyte during samples storage and processing procedures, in accordance with the USA Food and Drug Administration (FDA) bioanalytical method validation guidance.

#### 3.6.1. Selectivity

The selectivity of the method was evaluated by comparing the chromatograms of six different batches of blank plasma samples, plasma samples spiked with the mangiferin, neomangifer, timosaponin BII, anemarsaponin BIII, timosaponin AIII and IS, and plasma samples obtained from rats administered BZT.

#### 3.6.2. Linearity and LLOQ

The calibration curves were determined by plotting the peak area ratio (y) of analytes to IS versus the nominal concentration (x) of analytes with weighted (1/x^2^) least square linear regression. The lower limits of quantitation (LLOQ) of the assay were defined as the lowest concentration on the standard curve that can be quantitated with accuracy within 20% bias of the nominal concentration and RSD.

#### 3.6.3. Accuracy and Precision

Intra-day accuracy and precision of the assay were evaluated by analyzing QC samples at three concentration levels with six determinations per concentration on the same day and on three consecutive validation days. The accuracy was determined as RE (%) within 85%–115% from the nominal values, and the precision as RSD (%) within ±15% except for LLOQ, where it should be within 80%–120% for accuracy and not exceeding 20% of precision.

#### 3.6.4. Recovery and Matrix Effect

The recovery and matrix effect at three QC concentrations were determined in sets of five replicates. The extraction recoveries was calculated by comparing the peak responses of three QC samples with the responses of analytes from standard solutions spiked in post-extracted blank plasma at the same concentrations. Matrix effect was measured through comparison of the peak responses obtained from samples where the extracted matrix was spiked with standard solutions to those obtained from neat standard solutions at equivalent concentrations.

#### 3.6.5. Stability

The stability experiments were measured by analyzing replicates (n = 5) of three QC samples during the sample storing and processing procedures. For all stability experiments, freshly prepared stability testing QC samples were evaluated by using freshly prepared standard curve for the measurement. The post-preparation stability was tested by determined of the extracted QC samples stored in the auto-sampler (4 °C) for 24 h. The freeze and thaw stability were determined after three freeze-thaw cycles (−80 °C to room temperature). Long-term stability in rat plasma stored at −80 °C was studied for a period of one month.

### 3.7. Pharmacokinetic Study

Male Sprague-Dawley rats (230–280 g) were obtained from Shanghai SLAC Laboratory Animal Co., Ltd. (Shanghai, China) under license number SCXK 2007-0005. All animals were kept in an environmentally breeding room (temperature: 20–25 °C, humidity: 55%–65%) for 1 week before the experiments started. Animal welfare and experimental procedures were strictly in accordance with the Guide for the Care and Use of Laboratory Animals [19] and the related ethics of Nanjing University of Chinese Medicine. All rats were fasted for12 h with free access to water prior to the experiment and twelve rats were divided into two groups. The one group was administered into an oral dose of 10 g/kg BZT (65.5, 25.1, 103.7, 7.89, 19.2 mg/kg of neomangifer, mangiferin, timosaponin BII, anemarsaponin BIII and timosaponin AIII). The other group was administered an oral dose of 10 g/kg ZME (65.1, 24.9, 103.1, 7.92, 18.7 mg/kg of neomangifer, mangiferin, timosaponin BII, anemarsaponin BIII and timosaponin AIII). About 400 µL blood samples were collected into a heparinized tube via the orbital vein before drug administration and at 5, 15, 30, 45, 60, 90, 120, 240, 360, 480, 720, 1440 min after a single oral administration. The blood samples were immediately transferred to heparinized tubes and centrifuged at 3000 rpm for 10 min, and the supernatant was transferred into 2.0 mL Eppendorf tubes and stored at −80 °C until analysis. Blank plasma was obtained from the rat without oral administration and was used to investigate the assay development and validation.

### 3.8. Pharmacokinetic and Data Analysis

To calculate the pharmacokinetic parameters of analytes in BZT and ZME group, concentrations–time dada were analyzed by DAS 3.2 software (Mathematical Pharmacology Professional Committee of China, Shanghai, China). Data were measured as the mean ± standard deviation (S.D.) with triplicate measurements. The identification of significances between different groups was executed with Student’s *t*-test. A *p* value < 0.05 was considered statistically significant (SPSS statistical software package, version 17.0, SPSS Inc., Chicago, IL, USA).

## 4. Conclusions

In this paper, a sensitive, reliable and robust LC-MS/MS analysis method to simultaneously determine steroid saponins and flavonoids in rat plasma was successful validated. A comparative study on their pharmacokinetic parameters was performed to examine the Zhimu compatibility of Baihe. The results indicated that when BZT and ZME were administered orally to rats, the component of Baihe in BZT could influence the pharmacokinetic parameters of the steroid saponins but not influence the pharmacokinetic parameters of the flavonoids in Zhimu. These pharmacokinetic results could provide useful information for further research into the clinical pharmacokinetics of TCMs.
